# The interaction between resilience framework and neuron-astrocyte-synapse dynamics in AD

**DOI:** 10.3389/fnagi.2025.1644532

**Published:** 2025-09-11

**Authors:** Hongyue Ma, Haizhen Zhao, Xinhong Feng, Fengli Gao

**Affiliations:** ^1^Department of Neurology, Beijing Tsinghua Changgung Hospital, School of Clinical Medicine, Tsinghua Medicine, Tsinghua University, Beijing, China; ^2^Department of Nursing, Beijing Tsinghua Changgung Hospital, School of Clinical Medicine, Tsinghua Medicine, Tsinghua University, Beijing, China

**Keywords:** cognitive reserve, brain reserve, brain maintenance, astrocytes, Alzheimer’s disease

## Abstract

The concept of resilience can be used to explain why there are differences in the degree to which the brain functions of different individuals are impaired due to aging and pathological factors associated with neurodegenerative diseases. It encompasses cognitive reserve, brain reserve, and brain maintenance. Long-term research has identified a default mode network (DMN) related to cognitive reserve. This mode can modulate the negative impact of Alzheimer’s disease (AD) pathological burden on cognitive performance. Meanwhile, the association between neurons and glial cells plays a crucial role in the strength of neural network connections. Glial cells are widely distributed in the brain and interact closely with neurons. Among them, astrocytes are essential for maintaining the normal functions of the central nervous system. In both healthy and diseased states, astrocytes perform a variety of functions, including participating in the regulation of synaptic plasticity, synaptogenesis, maintaining glutamate and ion homeostasis, participating in cholesterol and sphingolipid metabolism, and being able to respond to environmental factors. All of these functions are associated with Alzheimer’s disease. In this review, first, we provided an overview of Cognitive Reserve, Brain Maintenance, and Brain Reserve. Then, we expounded on the possible mechanisms of action related to glial cells. Finally, we described their roles in Alzheimer’s disease and therapeutic development. This review may provide information and relevant therapeutic strategies for future research as well as the design of diagnostic and therapeutic interventions.

## 1 Introduction

Dementia is a comprehensive disorder characterized by the core feature of cognitive decline in patients, accompanied by significant impairment in activities of daily living, behavioral changes, and psychiatric abnormalities (Lopez et al., 2019). Multiple diseases can trigger dementia, including Alzheimer’s disease (AD), frontotemporal dementia, vascular dementia, dementia with Lewy bodies, etc. Among these causes, AD is the most dominant factor leading to dementia, accounting for approximately 50–70% of all dementia cases (van der Kant et al., 2019). AD is characterized by typical biological features, mainly manifested as the continuous accumulation of amyloid-β (Aβ) plaques, neurofibrillary tangles composed of aggregated tau proteins, and neurodegenerative changes (Hansen et al., 2018; Jack et al., 2018; Uytterhoeven et al., 2025). In recent years, studies have found that some AD patients can resist the clinical progression of dementia symptoms during their lifetime despite obvious pathological changes in the brain. Therefore, some researchers have proposed that the pathological severity of AD does not always show a positive correlation with an individual’s cognitive function (Katzman et al., 1988; Han et al., 2019).

Specifically, the degree of pathological changes in the brains of some healthy individuals even exceeds that of AD patients, yet they do not show signs of cognitive impairment. Conversely, some individuals exhibit symptoms of cognitive impairment even with a relatively low level of AD pathology (Katzman et al., 1988; Han et al., 2019). Similarly, a study conducted on elderly people aged 70–103 years showed that approximately one-third of the elderly who did not have impaired neuropsychological scores during their lifetime met the pathological criteria of Alzheimer’s disease upon postmortem examination [Neuropathology Group of the Medical Research Council Cognitive Function and Ageing Study (MRC CFAS), 2001].

In response to the inconsistent phenomenon between the pathological level of AD and cognitive function impairment, researchers proposed the cognitive reserve (CR) hypothesis. To further validate this hypothesis, Vockert et al. (2024) analyzed the longitudinal data of 490 AD dementia patients participating in a multicenter observational study and verified this hypothesis across the continuous risk stages from normal cognition to AD.

The study used functional magnetic resonance imaging of the visual memory encoding task to measure brain function. Through multivariate moderation analysis, an activity pattern related to CR was identified. This activity pattern was mainly characterized by the suppression of the Default Mode Network (DMN) and the activation of the inferior temporal regions (including the fusiform gyrus), with key nodes of the DMN connected by white matter fiber tracts (Cole et al., 2012; Franzmeier et al., 2017). Therefore, understanding the anatomical structure of the DMN and the hierarchical organization of its neural circuits is of great significance for us to gain in-depth insights into the organization of brain circuits, the mechanisms by which they promote cognitive and emotional functions, and how the impairment of these circuits leads to psychopathological changes. In addition, understanding the brain activity pattern related to CR can also help develop personalized treatment plans for patients, thereby enhancing or maintaining their cognitive reserve and delaying cognitive decline.

The status of the concept of CR has always been controversial when compared with brain reserve (BR) (Satz et al., 2011) and the recently proposed brain maintenance (BM) (Nyberg et al., 2012; Nyberg and Pudas, 2019). The core issues of the debate cover the definitions and measurement methods of each concept, their discriminant validity, the underlying neural mechanisms, and their roles in guiding the formulation of intervention measures to mitigate the impact of cognitive aging on behavior.

Stern et al. (2023) proposed a framework that clearly defined the three concepts of CR, BR, and BM. Additionally, they introduced the term “resilience” to encompass all these concepts. These research findings emphasized that there are individual differences in the brain’s ability to withstand damage and achieve functional compensation, which reflects that brain development is influenced by both genetic and environmental factors. Through methods such as functional training, education, participation in intellectual activities, and socio-cultural activities, and leveraging neural synaptic plasticity and systemic factors, the brain’s resistance to aging and neuropathological changes can be enhanced (Kittner et al., 1986; Zhang et al., 1990; Ahangari et al., 2023; Verkhratsky and Zorec, 2024). This review aims to comprehensively elaborate on the roles played by cognitive reserve, brain maintenance, and brain reserve in the nervous system, delve deeply into the intricate communication connections between neurons and glial cells, as well as their collaborative mechanisms during synapse formation. Meanwhile, it details the possible associations between glial cells and Alzheimer’s disease within the scopes of cognitive reserve, brain maintenance, and brain reserve, in the hope of providing valuable references for future clinical practice.

### 1.1 The complexity of CR, BR, and BM

The concept of reserve was proposed to explain individual differences in susceptibility to age-related brain changes and pathological alterations, such as those occurring in AD. Reserve can act as a regulator between pathology and clinical outcomes, thereby accounting for this discontinuity (Stern, 2012). BM, on the other hand, is a determinant of cognitive preservation in the elderly and plays a role at different stages of the life cycle. Therefore, understanding the framework of cognitive reserve, brain maintenance, and brain reserve can help us analyze and intervene in those factors that are crucial for maintaining brain health.

### 1.2 CR: an active compensation effect

Conceptually, cognitive reserve defines an individual property of the brain to maintain cognitive abilities above those expected given age or disease-induced damage. Fundamentally, cognitive reserve is the outcome of the history of each brain’s interaction with environmental factors throughout life, often referred to as the exposome, leading to either beneficial (lifelong learning, plasticity) or harmful (accumulation of pathological changes) alterations. These interactions also depend on the genes encoding an individual’s brain. These modifications are both neural tissue-based and systemic, with the closest interrelationship existing between the two.

The cognitive reserve model postulates the flexibility and adaptability of cognitive/brain networks, enabling the brain to actively resist the effects of age- or disease-related changes (Stern, 2002). Focusing on function, plasticity, and adaptability, cognitive reserve can be regarded as the “software” for computations in the brain, which is influenced by all aspects of life experiences (Stern, 2009) (Table 1).

**Table 1:** The definitions, neural correlations and measurement methods of CR, BR and BM.

Type	Definition	Component	Measurement method			Hypothesis	Neural correlation	References
			Brain changes	Cognitive change	Factors promoting CR, BR, BM			
Cognitive reserve	Cognitive reserve defines an individual property of the brain to maintain cognitive abilities above those expected given age or disease-induced damage.	1. Measure life-course-related brain changes, insults, diseases, or potential risk factors affecting cognitive outcomes. 2. Measure relevant cognitive changes. 3. Variables influencing the relationship between 1 and 2.	Structural MRI measurements of age-related changes.	Measurement of episodic memory at least two time points.	Measures related to years of education and lifestyle.	The CR hypothesis: factors related to CR will modulate the relationship between changes in multimodal MRI-based measurements and changes in episodic memory.	Molecular level (oxidative stress/calcium homeostasis/epigenetics); Cellular level (mitochondria/waste clearance/stem cells); Systemic level (synapses/neural connections/inflammation/stress signaling).	Stern et al. (2019)
Brain reserve	1. It is used to reflect the neurobiological state of the brain (the quantity of neurons, synapses, etc.) at any point in time. 2. Unlike CR, BR does not involve the active adaptation of functional cognitive processes in the presence of damage or disease.	1. Measurements of theoretically cognitive-related brain characteristics. 2. Relevant cognitive measurements.	It is estimated by intracranial volume (ICV) and measured quantitatively, including the number of neurons or synapses/dendritic spines.	Individuals who start at the same cognitive level may show the same rate of age – or disease-related decline in cognitive ability.	Measures related to multi-year education and lifestyle	–	BR is associated with individual differences in cognitive levels in the context of a specific amount of brain changes, damage, or diseases (such as amyloid plaques and neurofibrillary tangles). This association can be based on a threshold model, in which the consumption of a specific amount of neurobiological capital leads to disease-related changes.	Stern et al. (2019) and Uchida et al. (2022)
Brain maintenance	It refers to the relatively unchanged neural resources or the progression of neuropathological changes over time, which serves as a determinant for cognitive preservation in old age.	1. Measure the age-related brain changes, damage, or diseases that theoretically affect cognitive outcomes. 2. Measure the relevant cognitive changes. 3. Hypothetical variables that influence item 1.	Structural MRI measurements of age-related changes.	Episodic memory measurement at least two time points.	Measures related to years of education and lifestyle.	BM Hypothesis: Factors that promote BM will be associated with age-related changes in brain structure and/or function, thereby preserving episodic memory.	BM and CR are complementary concepts. BM explains the individual differences in cognitive trajectories related to the differences in the rate of brain changes. In contrast, CR addresses the individual differences in cognitive trajectories while controlling for neural resources or neuropathological changes.	Stern et al. (2019)

Evidence supporting cognitive reserve includes epidemiological data on lifestyle and clinical outcomes. Individuals with higher pre-morbid IQ, educational or occupational achievements, or those who engage in late-life leisure activities have a reduced risk of dementia and may experience a slower rate of age-related cognitive decline. A meta-analysis involving 29,000 people showed that compared with those with low cognitive reserve, individuals with high cognitive reserve had a 46% lower risk of developing dementia (Valenzuela and Sachdev, 2006).

Moreover, the theory of cognitive reserve acknowledges individual differences, which can enable some individuals to cope better with brain pathological changes than others. Thus, even in the presence of neuropathology, cognitive decline can be delayed (Valenzuela and Sachdev, 2006). Consequently, individuals with higher cognitive reserve are better able to handle brain changes and maintain higher levels of function despite brain deterioration (Stern, 2012; Stern et al., 2019).

### 1.3 BR: a passive mechanism

BR can be used to reflect the neurobiological state of the brain (the number of neurons, synapses, etc.) at any point in time. Unlike CR, BR does not involve the active adaptation of functional cognitive processes in the presence of damage or disease.

The brain reserve model defines reserve as a physical characteristic: some people have larger brains with more neurons and synapses, which may enable their brains to absorb more damage before cognitive function is affected. Therefore, brain reserve can be regarded as an individual difference in the brain’s “hardware” (Satz, 1993; Stern, 2012; Stern et al., 2019). Brain reserve can be considered a passive entity. It only manifests obvious signs of damage when an individual’s capabilities fall below a critical threshold of brain substrate loss (Stern et al., 2019). Those who initially have a higher BR can tolerate more depletion before symptoms appear (Stern et al., 2023) (Table 1).

### 1.4 BM: an active protective mechanism

The BM refers to the relative stability of neural resources or the slow progression of neuropathological changes over time, which is a determining factor for cognitive preservation in the elderly. BM is influenced by various factors such as genes, gender, early-life experiences, or differential exposures. These factors can slow down or prevent brain changes associated with aging and diseases. The focus is on the changes occurring over time. Thus, BM can potentially serve as an indicator of minimal changes in brain markers related to aging or diseases while maintaining cognitive function (Stern et al., 2023).

Related studies suggest that BM is not only influenced by genetic factors. It can also be regarded as an active protective mechanism, representing the ability to proactively maintain the integrity of brain structure and function and delay the accumulation of age-related damage. Various cell types in the brain, such as astrocytes, actively and dynamically regulate the ionic composition of the interstitial fluid in the central nervous system through a series of specialized pumps and transporters to maintain ionic homeostasis (Li et al., 2024; Verkhratsky and Zorec, 2024) (Table 1).

## 2 Molecular mechanisms of CR, BR, and BM

The evolution of the nervous system follows the principle of functional specialization, gradually giving rise to a rapid information transmission network dominated by neurons and a homeostasis regulation system led by glial cells (Verkhratsky and Nedergaard, 2016). DMN holds a prominent position in the overall organization of the brain and the hierarchical architecture of its functional networks. The close association between neurons and glial cells plays a crucial role in determining the connectivity strength of neural networks.

Notably, during the process of aging, the atrophy of glial cells has been identified as a key factor contributing to the decline in reserve (Streit et al., 2004; Popov et al., 2023; Verkhratsky and Zorec, 2024). Therefore, it is particularly important to conduct in-depth exploration of the potential mechanisms of cognitive reserve at the cellular level. As a vast population of non-neuronal cells in neural tissue, glial cells play an indispensable and crucial role in supporting and ensuring the normal functions of neurons, thus being hailed as the “logistical force” of the nervous system. In light of this, further in-depth exploration of the mechanisms of action of cognitive reserve, brain maintenance, and brain reserve at the cellular level can provide novel reference solutions for clinical diagnosis and treatment.

### 2.1 Network mechanisms of CR: neuron–glia associations

**Figure 1:**
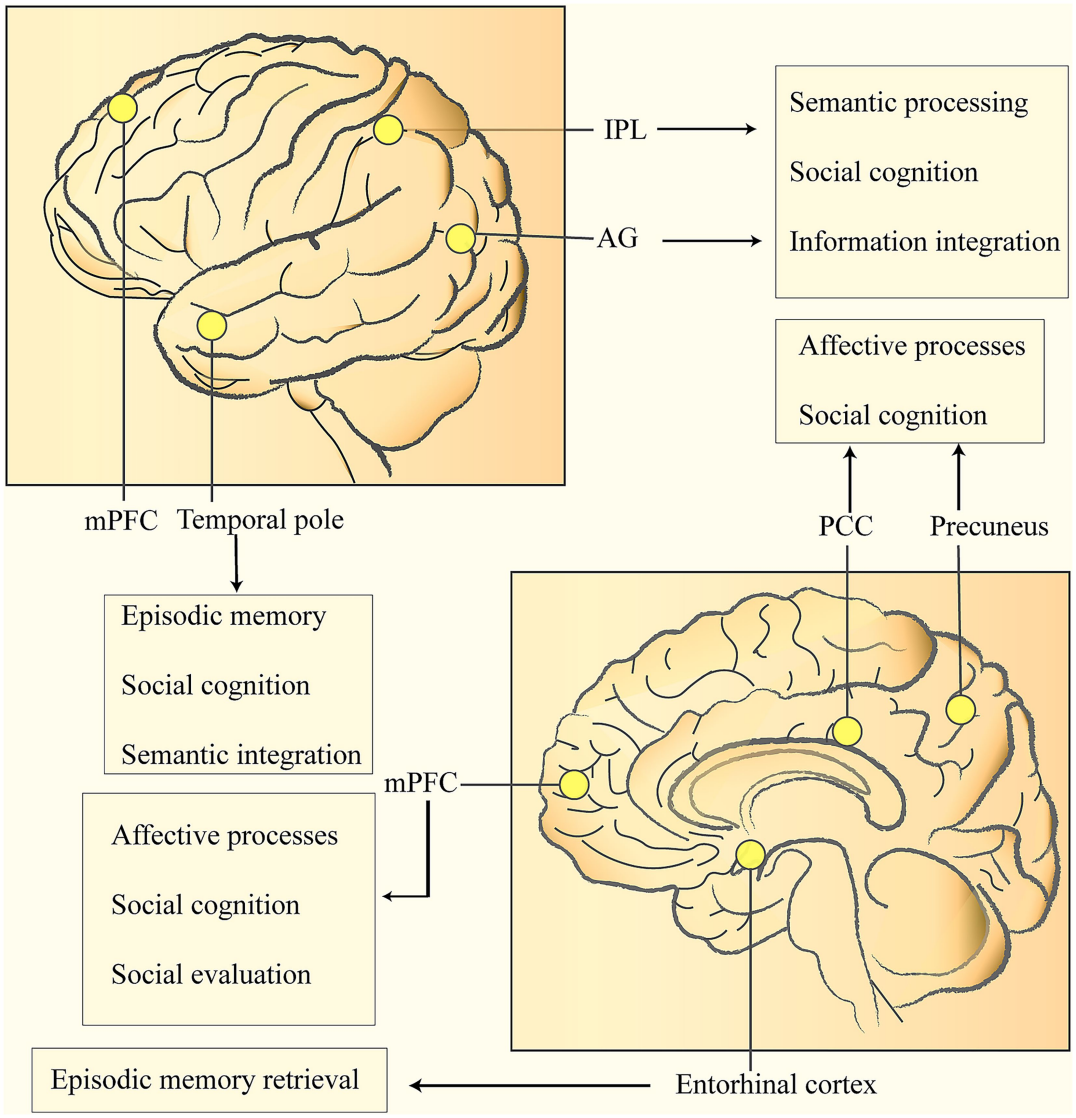
Nodes of the DMN related to cognitive reserve and their roles in social behavior: medial prefrontal cortex (mPFC), posterior cingulate cortex (PCC), bilateral inferior parietal lobules (IPL), temporal pole, etc. The IPL is located in the lower part of the parietal lobe, and the Angular Gyrus (AG) is situated at the posterior part of the inferior parietal lobule. Both play crucial roles in semantic processing, social cognition, and information integration. The PCC is connected to the mPFC via the cingulum bundle. The mPFC is located in the medial part of the prefrontal lobe (on both sides of the mid-line on the medial surface of the frontal lobe), and its main functions include affective processing, social cognition, and social evaluation. The PCC is located in the limbic lobe (the posterior one-third of the cingulate gyrus), and the Precuneus is in the middle of the medial surface of the parietal lobe. Both are involved in affective processing and social cognition. The Temporal pole is at the front end of the temporal lobe and is responsible for episodic memory, social cognition, and semantic integration. The Entorhinal Cortex is located in the medial part of the temporal lobe (the anterior part of the parahippocampal gyrus). As a key gateway to the hippocampal-limbic system, it is responsible for episodic memory retrieval.

The CR is mainly manifested by a more prominent expression of the task-active network, specifically demonstrated by the inactivation of the DMN and the activation of the inferior temporal regions including the fusiform gyrus (van Loenhoud et al., 2020; Stern et al., 2021). The DMN typically consists of a set of core brain regions and a set of supplementary brain regions. Its core brain regions include the medial prefrontal cortex (mPFC), the posterior cingulate cortex or precuneus, and the bilateral inferior parietal cortices; the supplementary brain regions include the medial temporal lobe and the temporal pole, etc. (Mars et al., 2012; Stern et al., 2018; Vockert et al., 2024) (Figure 1). Among them, the key sub-regions of the DMN, such as the left precuneus, the left posterior cingulate gyrus, the precuneus-cingulate gyrus conjunction area, and the medial frontal gyrus, have been proven to be closely related to CR (Cole et al., 2012; Franzmeier et al., 2017).

Related studies have found that the key nodes of the DMN exchange information through white matter fiber bundles (such as the cingulum bundle and the superior longitudinal fasciculus), thus forming an efficient network with small-world characteristics (Cole et al., 2012; Franzmeier et al., 2017). The extensively distributed nervous systems in the human brain support complex and flexible cognitive processes. These nervous systems are connected by white matter fiber bundles, which serve as supporting structures to enable information transmission and network integration between brain regions. Dynamic functional connectivity analysis shows that there is a hierarchical information processing pattern within the DMN: the anterior nodes are mainly responsible for leading self-related thinking, the posterior system focuses on scenario simulation, and the angular gyrus acts as a bridge for cross-modal information integration (Stern et al., 2005; Vockert et al., 2024).

In addition, the neural activity in the left prefrontal cortex (regardless of whether it belongs to the functional scope of the fronto-parietal network) and the global connectivity of the left frontal cortex are both significantly associated with CR. It is worth noting that existing evidence also suggests that the anterior cingulate cortex (ACC) may be involved in the regulation of the neural mechanism of cognitive reserve (Stern et al., 2005; Vockert et al., 2024).

Kahali et al. (2021) used quantitative Gradient-Recalled Echo (qGRE) MRI to map the cellular composition of the human brain and employed blood–oxygen level-dependent (BOLD) MRI to delve into the intrinsic relationship between brain cell components and the DMN.

The research findings indicated that within a single functionally defined unit of the network, the synchronization of connections between cell circuits defined by the BOLD signal mainly depends on the regional neuronal density. Moreover, the connection strength between functional units is also influenced by the glial and synaptic components in brain tissue cells. These mechanisms contribute to the widespread characteristics of the DMN.

Specifically, the visual network exhibits unique features. It has the highest neuronal density (while the glial cell and synaptic density are at the lowest levels), demonstrating the strongest BOLD signal consistency and the tightest internal network connections. In contrast, the DMN lies at the other end of the spectrum. Although it has relatively lower BOLD signal consistency, its cellular contents show a remarkable balance. This balance enables the DMN to play a highly prominent role in the overall organizational structure of the brain and the hierarchical functional network.

In conclusion, the association between neurons and glial cells plays a crucial role in the strength of neural network connections. In the network, the synchronous connections between cell circuits belonging to different functional units are not only closely related to the association between neurons in different functional units but also involve the interactions between neurons and synapses, as well as between neurons and glial cells.

### 2.2 Cellular mechanisms of BR: the role of glial cells

Brain reserve, initially defined as neural or functional reserve (Katzman, 1988), is regarded from an anatomical perspective as a resource determined by the brain size and/or structural characteristics when damaged. Initially, brain reserve was associated with brain volume. Interestingly, people with larger brains have a lower probability of developing dementia (Katzman et al., 1988; Schofield et al., 1997) or the quantity of neurons and synapses. The larger these values and the total brain volume are, the more capable an individual’s brain is of withstanding damage and demonstrating a higher reserve capacity (Katzman, 1988; Satz et al., 1993). Recent studies have shown that Stern et al. (2023) defined the Brain Reserve (BR) as a concept determined by a large number of neurons and the inter – neuronal connections formed by axonal projections (connectome) and synapses. It serves to reflect the neurobiological state of the brain at any given timepoint (the quantity of neurons, synapses, etc.). All of these are regulated by glial cells. Embryonic neurogenesis is a function of radial glial cells, while adult neurogenesis is carried out by radial glial stem cells, also known as neural stem cells, which exhibit all the major characteristics of astrocytes (Kriegstein and Alvarez-Buylla, 2009; Götz et al., 2016; Yeh et al., 2023) (Table 2).

**Table 2:** In the context of Alzheimer’s disease, the key molecules and possible mechanisms of action of astrocytes, oligodendrocytes, and microglia within the CR, BR, and BM frameworks.

Type	Astrocytes			Microglia			Oligodendrocytes			References
	Receptor	Cytokines, chemokines and other secreted mediators	Molecular mechanism	Receptor	Cytokines, chemokines and other secreted mediators	Molecular mechanism	Receptor	Cytokines, chemokines and other secreted mediators	Molecular mechanism	
Brain reserve	Glutamate transporters	Norepinephrine	The over-activation of glutamate receptors triggers an increase in the intracellular calcium ion concentration, affects synaptic plasticity, and ultimately leads to neurological dysfunction; abnormal Ca^2+^ signals in astrocytes caused by a decrease in norepinephrine levels.	PrP, NMDA receptor, P75NTR, mGluR5	TNFα, IL-1α, C1q	Imbalance of calcium ion homeostasis, inhibition of long-term potentiation (LTP), excessive phosphorylation of tau protein, mitochondrial dysfunction and oxidative stress.	–	MCT1	A decrease in the myelin level of oligodendrocytes and the blocked expression of MCT1.	Sun et al. (2018), Ma et al. (2022), and Zhang et al. (2024)
Brain maintenance	Glutamate transporters	Glutamate, ATP and γ-aminobutyric acid (GABA)	Neurons excessively release glutamate, triggering excitotoxicity.	Glutamate transporters, P2RY12	APOE, TGF-β, TMEM119	Glutamate accumulation in the synaptic cleft and excessive neuronal activity.	GABRA2, GABRB1, GRIA2, GRID2	NRXN1, NRXN3, APOE4	Impairment of pro-inflammatory and immune-related pathways, alteration of DNA damage response, abnormal lipid storage, and decreased myelination ability.	Santello and Volterra (2012), Grubman et al. (2019), Serrano-Pozo et al. (2021), Ma et al. (2022), and Zhang et al. (2024)

#### 2.2.1 Neuroglial cells and synaptic plasticity

**Figure 2:**
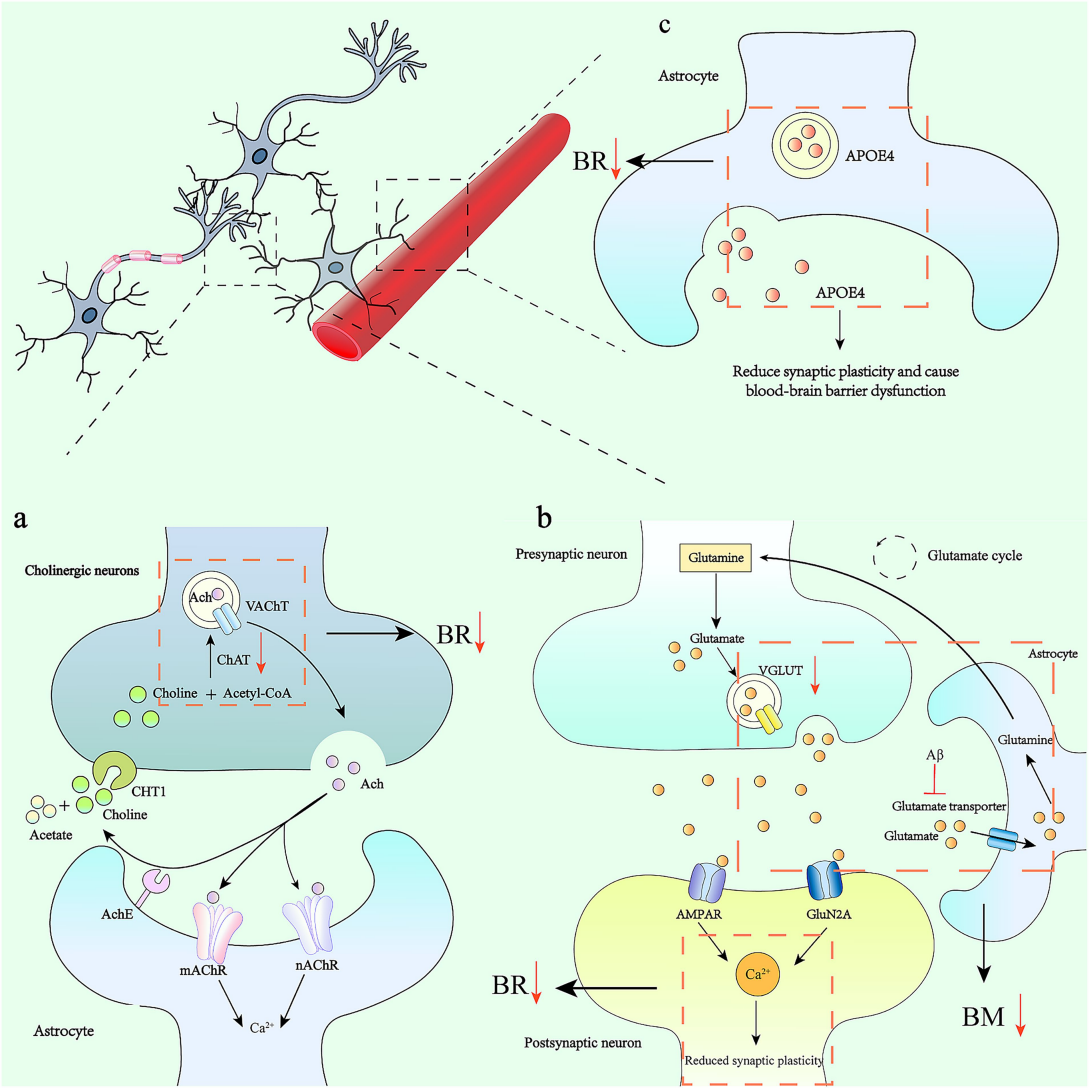
Astrocytes, synaptic plasticity and their alterations in Alzheimer’s disease. **(a)** The acetylcholine cycle and its effect on astrocytes: Acetyl-CoA and choline are synthesized into ACh under the action of choline acetyltransferase (ChAT), and are transported extracellularly in the form of vesicles. It binds to receptors on the surface of the postsynaptic membrane to complete signal transmission. In AD, there is a severe depletion of presynaptic cholinergic transmitters and a significant reduction in the activity of choline acetyltransferase. This affects synaptic plasticity, which in turn leads to impairments in learning, memory, and recognition functions. **(b)** The accumulation of amyloid-β (Aβ) in the brain regulates the uptake of glutamate by astrocytes through inhibiting glutamate transporters. This mechanism leads to the over-activation of glutamate receptors expressed on neurons, which in turn increases the intracellular calcium ion concentration (Ca^2+^), ultimately triggering neurological dysfunction. Meanwhile, calcium signals trigger astrocytes to release glutamate, which activates AMPA receptors, thereby exciting hilar interneurons. By activating the NMDA receptors of granule cells, it causes the depolarization of granule cells and promotes the formation of LTP at mossy fiber synapses. **(c)** APOE4 in astrocytes contributes to the reduction of synaptic plasticity and leads to blood-brain barrier dysfunction.

Synapses, as the crucial sites for information exchange and transmission between neurons, are of vital importance for the connection and function of neural circuits (Südhof, 2017). This process relies on synapse formation, elimination, and remodeling (Fourgeaud and Boulanger, 2007). In the initial stage, the axons of neurons extend to specific locations. Subsequently, protein assembly occurs at the axon terminals, leading to the formation of membrane proteins or vesicles. Then, the formed synaptic connections are strengthened through an activity-dependent manner of synaptic plasticity. Finally, immature or weakly functional synapses are pruned in an activity-dependent way (Fourgeaud and Boulanger, 2007; Huo et al., 2024). Glial cells play a crucial role in the process of synaptic plasticity, enabling precise regulation of individual synapses and neural circuits (Panatier et al., 2011; Stogsdill and Eroglu, 2017). The core function of synaptic plasticity is to provide a feedback mechanism for neural networks, allowing neurons to self-correct based on the effectiveness of signal transmission. When glial cells are absent, neurons form only a small number of synapses with weak connections (Ullian et al., 2001; Stogsdill and Eroglu, 2017). In the process of synaptic elimination, the main types of glial cells in the mammalian central nervous system—astrocytes, microglia, and oligodendrocyte precursor cells—all play important roles. Among them, synaptic pruning mediated by microglia includes processes such as physical contact, phagocytosis, and degradation of synapses (Auguste et al., 2022; Huo et al., 2024). Astrocytes are mainly involved in the regulation of synaptic plasticity and maintain cognitive function by releasing glutamate (Lee et al., 2022). Moreover, astrocytes mainly participate in the regulation of synaptic plasticity and maintain cognitive function by releasing glutamate (Lee et al., 2022). Moreover, this mediating function of astrocytes has also been confirmed in other hippocampal circuits. Astrocytes in the hilar region of the hippocampus act as a relay hub between cholinergic inputs and hippocampal granule cells. Acetylcholine activates the intracellular calcium signals of hilar astrocytes through nicotinic acetylcholine receptor (nAChR) and muscarinic acetylcholine receptor (mAChR). The calcium signals trigger the release of glutamate from astrocytes (either through a vesicular mechanism or bulk release). By activating AMPA receptors, this excites hilar interneurons; by activating the NMDA receptors of granule cells, it causes the depolarization of granule cells, promoting the formation of long-term potentiation (LTP) at mossy fiber synapses (Stogsdill and Eroglu, 2017; Lee et al., 2022), and further improving memory formation (Figure 2).

Cholesterol is one of the most important molecules in the synaptic membrane. Astrocytes are the main cells responsible for cholesterol synthesis in the brain. Cholesterol is synthesized in the endoplasmic reticulum of astrocytes in an ATP-dependent manner and is rapidly shuttled to the plasma membrane through a vesicle- and protein-mediated transport system. At the same time, astrocytes also synthesize lipoproteins and apolipoproteins for cholesterol transport. Cholesterol can be transported to neurons via ApoE, a lipid carrier synthesized by astrocytes in the central nervous system, to support the formation and function of synapses (Ferris et al., 2017).

#### 2.2.2 Astrocytes and neural regeneration

In the adult brain, neural stem cells are present in the subventricular zone of the central nervous system and the dentate gyrus of the hippocampus. These neural stem cells possess the ability for continuous self-renewal and can differentiate into specific neurons and glial cells at appropriate times, thereby replacing damaged neurons and repairing injured brain tissue.

Sirko et al. (2013) found that *in vitro*, the trans – differentiation of astrocytes into neurons depends on the stimulation of invasive injury and the action of the key signaling molecule Sonic hedgehog (SHH). Together, they constitute the necessary conditions for the occurrence of this *trans* – differentiation process. *In vivo* experiments have also confirmed that under the induction of specific brain injuries, astrocytes in the striatum can transdifferentiate into neurons (Magnusson et al., 2014). This indicates that astrocytes share similarities with neural stem cells in terms of their neural regeneration functions.

During adulthood, after brain injury, neural stem cells around the ventricles can migrate to the injured area and differentiate into specific neurons in a directed manner, thus promoting neural regeneration (Arvidsson et al., 2002). Astrocytes are widely distributed in the nervous system. When neurons are damaged or degenerate, astrocytes can become activated and exhibit good plasticity. Therefore, astrocytes have become important candidate cells for neuronal reprogramming.

In recent years, studies in relevant animal models have found that astrocytes can be reprogrammed into neural progenitor cells or even mature neurons. However, the efficiency of astrocytes transdifferentiating into neurons is relatively low. The functional integrity of the generated neurons and the extent to which astrocytes affect cognitive reserve still need further exploration.

As a star-shaped “companion” cell, astrocytes have recently been found to finely regulate communication between neurons. In particular, they can respond to norepinephrine, a signaling molecule that primes the brain and body for action. This causes astrocytes to release chemicals that ultimately act on neurons.

Norepinephrine-releasing neurons, which use norepinephrine as a neurotransmitter, are mainly distributed in the central nervous system and play a role in regulating various aspects such as alertness, attention, mood, and memory (Verkhratsky and Zorec, 2024). Studies have found that a reduction in the innervation of these neurons is directly associated with a decline in cognitive abilities. Therefore, enhancing the function of the noradrenergic system can increase BR.

Firstly, astrocytes are the main target cells of norepinephrine and possess Monoamine oxidase B (MAO-B), a central enzyme in catecholamine catabolism. In neurodegenerative diseases, the expression of MAO-B increases, which limits the function of the noradrenergic system. The use of MAO-B inhibitors can effectively increase norepinephrine levels and enhance BR (Park et al., 2019).

### 2.3 Cellular mechanisms of brain maintenance: the role of neuroglial cells

The maintenance mechanisms of the brain keep all substances in a stable state close to equilibrium. This state was defined by Claude Bernard as the stability of the internal environment (Duchesneau, 2024). The homeostasis of the central nervous system (as well as all other tissues, organs, and the entire organism) is not static but is achieved through continuous adjustments in response to environmental challenges, which is referred to as allostasis (Verkhratsky and Zorec, 2024).

Microglia are resident immune cells in the brain that maintain homeostasis. In a healthy brain, they perform homeostatic functions, which are characterized by the expression of transforming growth factor-β (TGF-β), transmembrane protein 119 (TMEM119), and P2RY12 (Butovsky et al., 2014; Krasemann et al., 2017). However, in response to brain injury or neurodegenerative diseases, reactive microglia lose their homeostatic molecular characteristics and functions (Paolicelli et al., 2022; Wang et al., 2024). Previous studies have shown that microglia play a role in physically monitoring the microenvironment through the interaction between ligands and receptors (Favuzzi et al., 2021; Huo et al., 2024). Research indicates that microglia express a variety of neurotransmitter receptors, including metabotropic and ionotropic glutamate receptors, gamma – aminobutyric acid (GABA) receptors, dopamine receptors, norepinephrine receptors, cannabinoid receptors, acetylcholine receptors, as well as some neuropeptide receptors and neuromodulator receptors such as adrenergic receptors and purinergic receptors (Liu et al., 2016; Huo et al., 2024) (Table 2).

Among them, glutamate, an important neurotransmitter in the brain, can be released not only from synaptic vesicles but also from glial cells. Excessive release of glutamate can stimulate the glutamatergic receptors on microglia, leading to the activation of microglia, the production of reactive oxygen species, and the release of ATP and cytokines. Consequently, this has corresponding effects on the reduction of dendritic spines and synaptic loss (Huo et al., 2024).

Astrocytes are the major homeostatic cells in the central nervous system and play a dominant role in brain maintenance. Astrocytes dynamically regulate the ionic composition of the interstitial fluid in the central nervous system through a series of specialized pumps and transporters, which is known as ionic homeostasis (Untiet et al., 2023; Rose and Verkhratsky, 2024). They control all major biological ions (such as sodium, calcium, potassium, and chloride) as well as trace elements that are crucial for the function of the central nervous system (MacAulay, 2020; Li et al., 2024; Verkhratsky and Zorec, 2024).

Astrocytes regulate ion concentrations by recycling neurotransmitters. High-affinity glutamate transporters uptake the neurotransmitter glutamate from the synaptic cleft for recycling (Anderson and Swanson, 2000; Sun et al., 2018). Astrocytes undertake the major task of glutamate transport. Glutamate is transported into the cytoplasm of astrocytes by binding to glutamate transporters, which are co-transported with Na^+^ into astrocytes. Na^+^ is then transported out of the cell by the Na^+^-K^+^-ATPase. Glutamate reacts with glutamine synthetase (GS) to form glutamine. The ATP consumed in this process is presumably supplied by the ATP generated through glycolysis. Notably, GS is only expressed in astrocytes (Parpura et al., 2017; Ma et al., 2022). Therefore, the glutamate-glutamine cycle plays a key role in maintaining the glutamate level in the central nervous system.

The generated glutamine is released into neurons. Then, glutamine enters neurons through the SLC1A5 receptor. The glutamine absorbed by neurons is converted into glutamate under the action of glutaminase (GLS), and it can also produce GABA through the action of glutamate decarboxylase (Ma et al., 2022).

**Figure 3:**
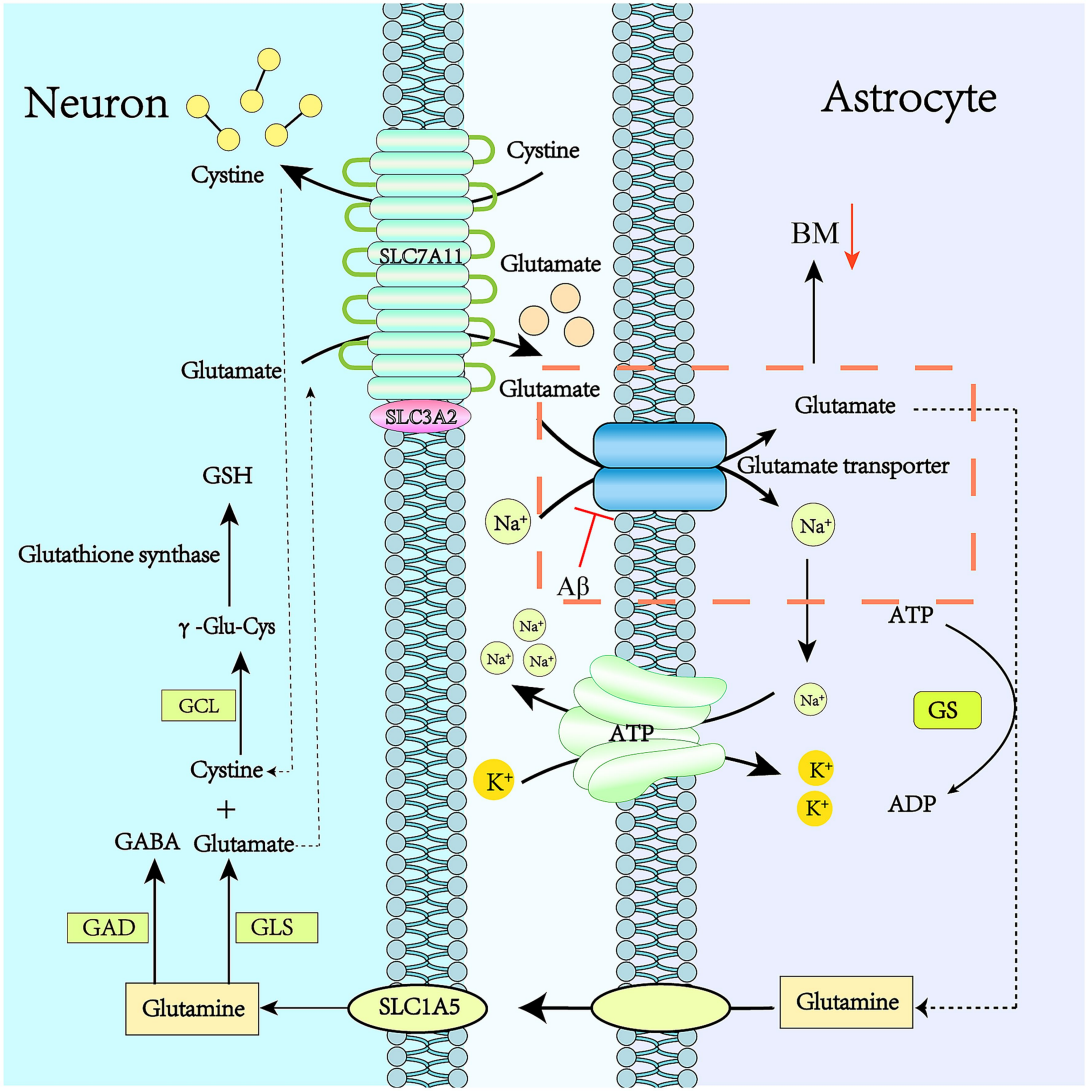
Glutamate recycling through the glutamate-glutamine cycle and its changes in the resilience framework of Alzheimer’s disease. Glu transporters are mainly distributed in astrocyte synapses. Glu binds to transporters which deliver it to the astrocyte cytoplasm. Glu transporters are co-transported into astrocytes by Na^+^ and Glu, and Na^+^ is transported to the extracellular space by Na^+^/K^+^-ATPase. Glu reacts with GS to produce Gln, and the ATP consumed in this process may be supplied by glycolysis. The resulting Gln is released into the neuron, and then Gln enters the neuron through the SLC1A5 receptor. The Gln absorbed by the neuron is converted into Glu under the action of glutaminase. GABA can also be produced by the action of Glu decarboxylase.

Another important role of glutamate in astrocytes is the synthesis of glutathione (GSH), which is crucial in the cell’s antioxidant defense and detoxification processes, as shown in Figure 3. Under high-glucose conditions, the amino acid metabolism of astrocytes is disrupted, the uptake of glutamate and the activity of GS are altered, and the level of GSH is reduced, which may induce cognitive dysfunction (Ma et al., 2022).

Therefore, as a crucial component of neurotransmission, astrocytes play multiple roles. They clear neurotransmitters (astrocytes express transporters to remove glutamate, γ-aminobutyric acid, catecholamines, and adenosine), carry out neurotransmitter catabolism (astrocytes convert glutamate to glutamine and degrade catecholamines and adenosine), and supply essential neurotransmitter precursors such as glutamine or L-serine to neurons (Verkhratsky et al., 2016; Verkhratsky and Zorec, 2024). Astrocytes store glycogen, and the intracellular breakdown of glycogen can produce L-lactate. L-lactate can provide energy support for neurons and is an important fuel source for them (Yellen, 2018; Fink et al., 2021; Rae et al., 2024). Similarly, oligodendrocytes support axons (Simons and Nave, 2016), while oligodendrocyte precursor cells (OPCs) provide lifelong myelin regeneration and activity-dependent formation of new myelin sheaths, also contributing to the overall homeostasis of the central nervous system (Yi et al., 2023). Finally, microglia are involved in debris clearance, continuous repair, and maintaining the immune homeostasis of neural tissue (Stratoulias et al., 2019).

## 3 CR, BR, and BM in relation to Alzheimer’s disease

Understanding CR, BR, and BM can help us comprehend the brain’s ability to resist damage. Among these, glial cells are the main elements for maintaining homeostasis and providing defense in the central nervous system. Aging is associated with a decline in cognitive abilities and is a major risk factor for neurodegenerative diseases. By understanding and harnessing the functions of glial cells and adopting appropriate strategies to enhance cognitive reserve, we may be able to better safeguard brain health and delay the decline of cognitive abilities.

### 3.1 The network mechanism of CR: the association between the DMN and Alzheimer’s disease

In AD, the brain regions most frequently involved in alterations of functional connectivity are those within the DMN, which are considered to be selectively vulnerable to AD neuropathology (Warren et al., 2013). From a neurobiological perspective, poor DMN connectivity is thought to be the result of activity-dependent tau propagation, from the medial temporal lobe to densely connected cortical hubs (Sperling et al., 2009; Huijbers et al., 2019; Maass et al., 2019; Vockert et al., 2024). Vockert et al. (2024) found that changes in DMN effective connectivity mediated the association between social isolation and the incidence of dementia. When examining the connectivity differences between cases and controls, the researchers found an increased inhibitory tone from the prefrontal and parietal cortices to the medial temporal lobe, as well as a decreased inhibitory tone from the medial temporal lobe to the prefrontal cortex. The increased inhibitory tone may potentially reflect homeostatic compensation to maintain the excitatory-inhibitory balance within the network. Due to the neuronal silencing effect of tau accumulation in the medial temporal lobe in the early stages of the disease, the inhibitory connections from the parahippocampal formation to the prefrontal cortex are weakened.

Therefore, an important direction for future research is to collect longitudinal imaging data as well as data on subjective and cognitive impairments, in order to understand the clinical significance of different connections in the connection patterns of the brain’s DMN. DMN dysconnectivity may be better understood in the context of a broader dynamics involving other long-range networks. In high-risk populations for AD, both the salience network and the frontal parietal lobe control network are altered, and these changes are associated with future cognitive decline. Incorporating these networks into the development of future effective connectivity-based prediction models is of great significance.

### 3.2 The cellular mechanisms of BR: association between glial cells and Alzheimer’s disease

The BR reflects the brain’s capacity to withstand risks in terms of its volume and structure. A larger brain volume indicates a greater number of neurons and synaptic connections, as well as a stronger ability to resist cognitive decline. The interaction between neurons and glial cells promotes the formation and remodeling of synapses. In the absence of glial cells, neurons form weaker synapses (Pannasch et al., 2011; Kahali et al., 2021). The close relationship between neurons and glial cells plays a crucial role in establishing strong network connections. This is consistent with the role of glial cells in supporting neurons by providing metabolic and regulatory functions (Poskanzer and Yuste, 2011; Suzuki et al., 2011; Magistretti and Allaman, 2015; Kahali et al., 2021), as well as in supporting neurite growth and neuronal guidance (Ullian et al., 2001; Pannasch et al., 2011). As people age, astrocytes and microglia also show significant atrophy in the elderly brain, which leads to a decline in homeostatic support and defensive capabilities (Streit et al., 2004; Popov et al., 2023; Verkhratsky and Zorec, 2024). Aging is also associated with tau astrogliopathy, a disease that serves as the basis for various neurodegenerative pathologies related to cognitive impairment (Schultz et al., 2000; Kovacs et al., 2016; Verkhratsky and Zorec, 2024).

Astrocytes take up the neurotransmitter glutamate from the synaptic cleft using high – affinity glutamate transporters for recycling (Duan et al., 1999; Anderson and Swanson, 2000; Lee et al., 2022). When the glutamate transport function of astrocytes is impaired, neurons will over – release glutamate, leading to excitotoxicity and subsequent neuronal death (Schousboe et al., 1997) (Figure 3). This process has long been regarded as a key part of the pathogenesis of neurodegenerative diseases (Doble, 1999; Pitt et al., 2000; Maragakis and Rothstein, 2004; Dong et al., 2009; Lee et al., 2022).

In addition, an increase in the intracellular Ca^2+^ level of astrocytes can stimulate the release of glutamate. The accumulation of Aβ in the brain inhibits glutamate transporters, affecting the uptake of glutamate by astrocytes. This causes excessive activation of glutamate receptors expressed on neurons, leading to an increase in intracellular calcium ion concentration, which affects synaptic plasticity and ultimately results in neurological dysfunction (Lee et al., 2022).

As the main target cells of norepinephrine, astrocytes have been found to exhibit abnormal Ca^2+^ signaling in mouse models of AD due to reduced norepinephrine levels (Åbjørsbråten et al., 2022). Using reversible or irreversible MAO-B inhibitors may be an effective strategy to enhance CR (Park et al., 2019). tDCS can improve memory, promote motor function recovery, relieve depression, and delay the progression of cognitive impairment in AD patients (Kuo et al., 2014). In addition, tDCS presents a promising avenue for activating norepinephrine – releasing neurons. Alternatively, the transplantation of norepinephrine – releasing neurons also stands out as a feasible strategy (Kuo et al., 2014).

Its mechanism of action is achieved through astrocytes and their adrenergic receptors. Exposure to tDCS triggers a large number of Ca^2+^ signals in astrocytes, and these signals can be blocked by ablation of noradrenergic neurons or pharmacological inhibition of α1-adrenergic receptors (Monai et al., 2016; Monai and Hirase, 2018).

In the amyloidosis model of APP/PS1 mice (6–8 months old), Adult oligodendrocyte precursor cells (aOPCs), near Aβ plaques exhibit increased proliferation and differentiation (Behrendt et al., 2012; Yi et al., 2023). In both AD patients and APP/PS1 mice, the immunoreactivity of aOPCs and NG2 near amyloid plaques is enhanced (Nielsen et al., 2013; Sun et al., 2018; Yi et al., 2023). In the postmortem human AD cortex, the total number of oligodendrocytes is reduced (Behrendt et al., 2012; Yi et al., 2023). In both AD patients and mouse models, the total amount of myelin decreases over time. It can be speculated that compensatory myelin regeneration may occur during the progression of AD. In this context, pharmacologically enhancing aOPC differentiation and myelin regeneration may emerge as a potential strategy to alleviate the cognitive impairment caused by AD. Relevant studies have shown that among various neurotransmitter receptors expressed by microglia, the activation of type III subtypes of metabotropic glutamate receptors (mGluR) has a neuroprotective effect, and their stimulation helps inhibit neuronal death. In contrast, type II subtypes promote the activation of neurotoxic microglia. Therefore, interventions targeting microglial receptors may provide new therapeutic strategies for delaying the progression of AD (Ghimire et al., 2025).

### 3.3 The cellular mechanisms of BM: association between glial cells and Alzheimer’s disease

Both astrocytes and oligodendrocytes (including those in fetal and adult stages) are vulnerable to oxidative stress, as well as glutamate and ATP excitotoxicity (Káradóttir et al., 2005; Matute et al., 2006; Giacci et al., 2018; Yi et al., 2023). Relevant studies have shown that the dysfunction of astrocytic glutamate transporters can lead to excessive glutamate release from neurons (Schousboe et al., 1997). This promotes neuronal death through excitotoxicity, a process that has long been associated with the occurrence of neurodegenerative diseases (Pitt et al., 2000; Maragakis and Rothstein, 2004; Dong et al., 2009; Soni et al., 2014). The research team led by Ma et al. (2022) found that the expression of glutamate transporters in the cerebral cortex of AD patients is decreased, and the decrease in vesicular glutamate transporter 1 (VGLUT1) is more pronounced. This may result in the failure to efficiently clear glutamate in the synaptic cleft, thereby triggering excitotoxicity. In fact, the pro-inflammatory cytokine tumor necrosis factor (TNF) increases glutamate production, thus triggering excitotoxicity and Ca^2+^ signaling. An increase in Ca^2+^ levels in glial cells leads to the release of glutamate, ATP, and gamma-aminobutyric acid (GABA). Additionally, the release of GABA and ATP by reactive astrocytes has been shown to be associated with the disease in Alzheimer’s disease models (Santello and Volterra, 2012). Dysfunctional glial-neuronal metabolic interactions are characteristic features of various neurodegenerative diseases (Sun et al., 2018).

In addition, glutamate accumulates in the synaptic clefts of the cerebral cortex in AD patients. In the presence of excessive glutamate and/or ATP, aOPCs rapidly die, which can lead to white matter damage and neurological symptoms (Deng et al., 2004; Salter and Fern, 2005; Matute, 2011). The death of aOPCs has also been observed in brain injuries associated with inflammation and neurodegeneration (Yi et al., 2023). In the human Alzheimer’s disease brain, pexin-B3-positive aOPCs are associated with extracellular Aβ_1–42_ deposits, suggesting that pexin-B3-positive aOPCs may be the cells that produce Aβ (Nihonmatsu-Kikuchi et al., 2021; Yi et al., 2023). Single-cell RNA sequencing shows that genes encoding GABA receptors (GABRA2 and GABRB1), glutamate receptors (GRIA2 and GRID2), and neurexins (NRXN1 and NRXN3) in aOPCs are downregulated. These genes are all related to behavior, cognition, and synaptic organization (Grubman et al., 2019). In aOPCs from postmortem Alzheimer’s disease brains, the presence of the apolipoprotein ε4 allele (APOE4, a genetic risk factor for Alzheimer’s disease) is associated with impaired pro-inflammatory and immune-related pathways, altered DNA damage responses, abnormal lipid storage, and decreased myelination ability (Yi et al., 2023; Blanchard et al., 2024). Selective deletion of APOE4 in astrocytes can rescue tau-mediated neurodegeneration (Blanchard et al., 2021; Tcw et al., 2022) (Figure 2). APOE4 also contributes to reducing synaptic plasticity and causing blood–brain barrier dysfunction (Bovolental et al., 1997; Tcw et al., 2022) (Table 2).

The latest research has discovered a disease-associated microglia (DAM) signature, namely a significant reduction of microglial homeostatic markers in DAM. In a mouse model of Alzheimer’s disease (AD), TMEM119, a microglia – specific marker, is significantly reduced during amyloid plaque formation and is closely related to the spatial distribution of microglia near Aβ plaques (Liu et al., 2025). Therefore, the underlying mechanisms driving the changes in microglial TMEM119 and its significance in the pathogenesis of AD need further investigation.

## 4 Cognition intervention-related therapies

Epidemiological evidence suggests that experiences at all stages, even those in later life, can enhance reserve. These findings support the possibility of interventions even in old age to strengthen reserve, slow down age-related cognitive decline, and extend healthy aging (Stern, 2012). Therefore, in the prevention and treatment of neurodegenerative diseases, CR, BR, and BM constitute an individual’s “triple defense system” against pathological damage. We will summarize from the above three aspects, from mechanisms to intervention strategies, to provide a reference for future cognitive intervention measures in the treatment of neurodegenerative diseases.

In terms of cognitive reserve, the disconnection hypothesis of dementia has received increasing support from neuroimaging and neurophysiological evidence, which indicates that there are changes in the functional connectivity of the brain in patients with cognitive impairment (Liu et al., 2014). DMN was initially described as a network of regions that were co-activated in the task-negative state during functional imaging studies. In other words, these brain regions seem to be more active when participants are at rest. However, research has shown that the DMN is involved in higher cognitive processes such as social cognition and mental time travel (Mars et al., 2012; Warren et al., 2013; Yeshurun et al., 2021), which has led to the contemporary view that the DMN provides individuals with a sense of their self-narrative (Menon, 2023). Cognitive training may improve brain function at the neural network level (Buonomano and Merzenich, 1998). Meanwhile, neuroimaging analysis has shown enhanced connectivity between the executive control network (ECN) and the DMN in patients, and there is a correlation between these two changes. This view has also been confirmed in a study on music rehabilitation therapy (Li et al., 2014). Therefore, cognitive interventions may exert their effects by enhancing the connectivity of the brain functional network in patients.

In terms of BR, enhancing neuronal plasticity, especially in the higher-order frontal, parietal, and temporal association cortices, may improve neural function and prevent the loss of typical AD neuronal processes. Impaired neural plasticity has been observed in early AD patients (Inghilleri et al., 2006; Battaglia et al., 2007; Terranova et al., 2013). In patients with mild cognitive impairment (MCI), an increase in synaptic plasticity is considered to reduce the Aβ burden, suggesting that there is a balance between synaptic activity-driven Aβ production and synaptic activity-driven degradation, with a tendency to clear extracellular Aβ and reduce its burden (Selkoe, 2008). Therefore, early treatment methods targeting synaptic plasticity to increase brain reserve may be a way to slow the progression of dementia symptoms or prevent AD. Drulis-Fajdasz et al. (2023) treated aged mice with the glycogen phosphorylase inhibitor BAY U6751 for 2 weeks. The results showed that this treatment could alleviate memory deficits in aged mice and stimulate neural plasticity, indicating that lactate produced by astrocyte glycolysis is crucial for synaptic plasticity and long-term memory. Related studies have shown that under hyperglycemic conditions, astrocyte glucose metabolism is disrupted, glucose uptake and utilization are reduced, and excessive glutamate secretion leads to neuronal synaptic dysfunction, which may induce cognitive impairment (Suzuki et al., 2011). Therefore, strict blood glucose control is very important for preventing cognitive decline and reducing the risk of mild cognitive impairment.

In terms of BM, when the synaptic function of neurons is dysregulated, the neurotransmitters and neuromodulators in the extracellular environment become imbalanced. Among numerous neurotransmitters, glutamate plays a crucial role (Verkhratsky and Zorec, 2024). It is not only the most important excitatory neurotransmitter in the brain but also deeply involved in the glutamate – glutamine cycle (Ma et al., 2022). This cycle is of great significance as it acts like a link, skillfully connecting the metabolic processes of glucose and amino acids with synaptic transmission, intracellular homeostasis, and cellular energy metabolism (Ma et al., 2022). Studying targeted therapeutic methods to reduce the excitotoxicity caused by glutamate is of great importance for the treatment of AD.

Baicalein, a natural polyphenolic compound, inhibits lipoxygenase by reducing oxidative stress and serves as an anti – inflammatory and neuroprotective agent. Its properties include inhibiting the depletion of GSH, the degradation of glutathione peroxidase 4 (GPX4), and lipid peroxidation; reducing the level of nuclear factor E2-related factor 2 (Nrf2); and inhibiting 12/15-lipoxygenase (12/15-LOX) (Xie et al., 2016; Li et al., 2019; Yuan et al., 2020). Feeding APP/PS1 mice with baicalein showed a decrease in the activity of beta-site amyloid-precursor protein cleaving enzyme 1 (BACE1), a reduction in the levels of Aβ and phosphorylated tau (p-tau), and better results in behavioral tests.

Another polyphenolic compound, curcumin, scavenges reactive oxygen species (ROS), increases the levels of superoxide dismutase (SOD), sodium – potassium adenosine triphosphatase (Na^+^ K^+^-ATPase), catalase, GSH, and mitochondrial complex enzymes (Ege, 2021), inhibits the aggregation of Aβ, and reduces the effect of phosphorylated tau protein. However, curcumin has poor water solubility and insufficient bioavailability in clinical trials, so its application in the clinical treatment of Alzheimer’s disease is limited (Tang and Taghibiglou, 2017; Reddy et al., 2018; Ege, 2021) (Table 3).

**Table 3:** Summary of the available cognitive intervention-related therapies for AD.

Type	Inhibitors	Mechanism of action	Experimental models	Effect	Effector cell	References
MAO-B inhibitor						
Reversible	KDS2010	Significantly inhibits the GABA production level in astrocytes and astrogliosis, and enhances synaptic transmission	APP/PS1	Improve the learning and memory impairments in APP/PS1 mice.	Astrocytes	Park et al. (2019)
Irreversibility	Selegiline	Inhibit the production of GABA by reactive astrocytes.	APP/PS1	It can improve the cognitive deficits of AD patients in the short term.	Astrocytes	Jo et al. (2014) and Park et al. (2019)
Non-invasive brain stimulation techniques						
rTMS	–	–	–	Induce and regulate human neural plasticity	–	Kuo et al. (2014)
tDCS	–	By generating subthreshold and stimulus polarity dependent membrane potential changes, the spontaneous firing frequency of neurons is regulated	G7NG817 mice	Anodal tDCS improves visual recognition memory.	–	Kuo et al. (2014), Monai et al. (2016), and Monai and Hirase (2018)
Glycogen phosphorylase inhibitor						
–	BAY U6751	Improve the formation of LTP in the CA1 region of the hippocampus	Aged mice	Reduce memory deficits in aged mice and stimulate neural plasticity	Hippocampal CA1 region	Drulis-Fajdasz et al. (2023)
Natural polyphenol compounds						
–	Baicalein	Inhibit GSH depletion, GPX4 degradation, and lipid peroxidation	APP/PS1mice, C57/BL6 mice, HT22 cells	Improve the cognitive dysfunction of patients with AD	Hippocampus	Xie et al. (2016), Li et al. (2019), and Xie et al. (2016)
–	Curcumin	Inhibit Aβ aggregation, reduce p-tau protein, possess antioxidant and anti-inflammatory effects	SH-SY5Ycells, APP/PS1mice,5 × − familial AD (5XFAD)	–	The CA1 region of the hippocampus	Tang and Taghibiglou (2017), Reddy et al. (2018), and Ege (2021)

In addition, brain – derived neurotrophic factor (BDNF) is an important neurotrophic factor that is expressed in multiple brain regions such as the hypothalamus, cerebral cortex, brainstem, and hippocampus. It plays a key role in the survival, differentiation, growth of dendrites and axons of neurons, and also in regulating synaptic plasticity (Pressler et al., 2015; Jeong et al., 2016). Some studies have shown that computer – based cognitive interventions can also increase the BDNF level in subjects (Pressler et al., 2015; Jeong et al., 2016). Therefore, the rational application of computer – based models is helpful for patients with cognitive decline and provides a reference for clinical rehabilitation.

## 5 Early diagnostic biomarkers for Alzheimer’s disease

In recent years, research on the early detection of dementia has tended to focus on biomarkers that can directly reflect the deposition of pathogenic proteins in AD, such as amyloid-β and tau proteins in cerebrospinal fluid. However, the predictive ability of these biomarkers in healthy populations is poor. In contrast, the plasma phosphorylated tau level has high predictability for AD neuropathology. When combined with cognitive and genetic data, it can relatively accurately predict the conversion rate from mild cognitive impairment (MCI) to AD. Differences in CR, BR, and BM abilities can affect the performance of these biomarkers. Individuals with higher CR, BR, and BM may still maintain good cognitive function after abnormal biomarker results, thus delaying the onset of symptoms (Ereira et al., 2024; Vockert et al., 2024).

Ereira et al. (2024) found that the effective connectivity in the default mode network (DMN) can serve as a non-invasive biomarker for predicting the future incidence of dementia, and it is superior to structural MRI data. Changes in DMN connectivity are associated with the polygenic risk of AD and social isolation, which may weaken CR, BR, and BM. Astrocyte-neuron connectivity also plays an important role in DMN function. Astrocytes can influence the connectivity and function of the DMN by regulating neurotransmitters and maintaining ion balance. When astrocyte-neuron connectivity is impaired, it may accelerate the impact of pathological proteins in the DMN, thereby affecting the occurrence and development of dementia.

Although fMRI has advantages in diagnosis and prognosis, it is costly and sensitive to head movement. Future research needs to evaluate its adaptability to low-quality data.

Oh et al. (2025) found that the ratio of synaptic proteins YWHAG: NPTX2 is expected to become a new biomarker. The ratio in cerebrospinal fluid increases with age and is closely related to the risk of AD progression. Changes in astrocyte-neuron connectivity may affect the expression and function of synaptic proteins, thus influencing this ratio. Blood-based detection methods have attracted attention due to their convenience. Horie et al. (2025) pointed out that the level of MTBR-tau243 in the blood can reflect the degree of tau protein aggregation in the brain and the progression of the disease. Combining resting-state functional magnetic resonance imaging with the detection of MTBR-tau243 in the blood is expected to improve the prediction accuracy of the future incidence of dementia. Meanwhile, considering factors such as CR, BR, BM, and astrocyte-neuron connectivity may help develop more effective individualized treatment plans for patients, which is worthy of further research.

## 6 Discussion

In recent years, research has revealed that the formation of CR and BR is not dominated by a single factor, but rather stems from the dynamic accumulation of multi-dimensional life experiences. This includes lifestyle optimization (such as a Mediterranean diet and regular exercise), continuous cognitive stimulation (education, complex occupations), and positive psychological traits (open personality, stress resistance). This discovery has overturned the traditional view of the “fixed reserve theory,” emphasizing the continuous regulatory role of neural plasticity throughout the life cycle. Even interventions in old age can still reshape the brain network and delay the pathological process of dementia.

From a practical application perspective, the concept of cognitive reserve can be incorporated into doctors’ diagnostic processes. It is worth noting that for patients with AD, a higher cognitive reserve is associated with a faster decline in the condition. This phenomenon implies that it is particularly crucial to take cognitive reserve into account when conducting clinical trials that rely on the differences in the decline rates of patients in the drug group and the placebo group.

In this field, neuroindicators directly measurable and related to cognitive reserve hold promise as early biomarkers for predicting dementia, such as specific patterns presented by functional magnetic resonance imaging (fMRI). The DMN exhibits some unique characteristics in fMRI imaging. On the one hand, it shows relatively low BOLD signal coherence and network connection strength. On the other hand, it demonstrates remarkable cellular circuit diversity. These seemingly contradictory characteristics precisely reflect the important position of the DMN in the overall organization of the brain and the hierarchical structure of functional networks (Kahali et al., 2021).

Notably, the individualized CR score constructed based on fMRI technology is of great significance. This score can not only mitigate the adverse effects of Alzheimer’s disease (AD) pathological changes on cognitive performance but also shows a positive correlation with the number of years of an individual’s education. This implies that the higher the level of education, the higher the individualized CR score based on fMRI is likely to be, and the stronger the cognitive resistance to AD pathology.

From a cell biology perspective, the signals secreted by astrocytes play a crucial role in the formation, maturation, and plasticity of neural circuits. Meanwhile, the neuron–glia interaction network, as a hub in the regulation of cognitive reserve, provides the basis for the brain to process information efficiently and maintain cognitive function. The synapses and connectome regulated by glia, as the structural basis of BR, have important regulatory effects on axon growth, synapse formation, and stability. The homeostatic regulation of astrocytes, as the core executor of BM, is essential for the normal function of neurons. This ionic homeostasis ensures the stability of physiological processes such as neuronal excitability, neurotransmitter release, and signal transduction.

Astrocytes have the potential to be reprogrammed into functional neurons. Due to the large number of astrocytes in the central nervous system, the reprogramming of astrocytes may contribute to the treatment of neurodegenerative diseases. In fact, studies have reported encouraging therapeutic effects of *in vivo* glia-to-neuron conversion in preclinical models of major neurodegenerative diseases such as Alzheimer’s disease, Parkinson’s disease, and Huntington’s disease (Wu et al., 2020; Lee et al., 2022). However, the conversion of glial cells into neurons is currently controversial. Using lineage tracing methods, some studies have shown that glial cells do not transform into neurons, but rather endogenous neurons are labeled (Hoang et al., 2021; Wang et al., 2021; Lee et al., 2022). Overall, these studies may highlight the potential of new regenerative medicine therapeutic interventions targeting astrocytes in the treatment of neurodegenerative diseases.

Based on all these findings, taking a comprehensive consideration of cognitive reserve, brain reserve, and brain maintenance allows us to stand at a more macroscopic and systematic perspective and deeply understand various conditions related to brain changes. This helps us to more accurately grasp the mechanisms of the brain in both healthy and diseased states, providing a solid foundation for the early diagnosis, intervention, and treatment of neurodegenerative diseases such as dementia.
